# Effect of CBC-Derived Inflammatory Indicators in Predicting Chronic Kidney Disease Risk in Hypertrophic Cardiomyopathy Patients

**DOI:** 10.3390/biomedicines13040997

**Published:** 2025-04-20

**Authors:** Changying Zhao, Luqin Yan, Yong Liu, Siyuan Chen, Beidi Lan, Ruohan Liu, Jinqi Xin, Tao Shi, Xiaohong Yang

**Affiliations:** 1 Department of Cardiovascular Surgery, The First Affiliated Hospital of Xi’an Jiaotong University, No. 277 Yanta West Road, Xi’an 710061, China; 2 Intensive Care Unit of Pulmonary and Critical Care Medicine, The Second Affiliated Hospital of Xi’an Jiaotong University, Xi’an 710114, China; 3 Xi’an Jiaotong University Health Science Center, Xi’an 710061, China

**Keywords:** hypertrophic cardiomyopathy, chronic kidney disease, CBC-derived inflammatory indicators, systemic inflammation response index, systemic immune inflammation index

## Abstract

**Background:** Hypertrophic cardiomyopathy (HCM) is a prevalent condition that often coexists with chronic kidney disease (CKD), significantly impacting patient prognosis. This study aimed to investigate the predictive value of complete blood cell counts derived inflammatory indicators in assessing CKD risk in HCM patients. **Methods:** This study enrolled HCM patients and categorized them into CKD and non-CKD group based on discharge diagnoses. Analyzed indicators included systemic inflammation response index (SIRI), systemic immune inflammation index (SII), neutrophil-to-lymphocyte ratio (NLR), and platelet-to-lymphocyte ratio (PLR). Least absolute shrinkage and selection operator (LASSO) logistic and multivariable logistic regression were employed to identified independent risk factors for CKD, which were subsequently utilized to develop a nomogram. **Results:** A total of 1795 HCM patients were included, including 112 (6.24%) individuals assigned to the CKD group. In univariate analyses, NLR (AUC: 0.755; 95%CI: 0.711–0.800) demonstrated superior accuracy compared to others. Eighteen baseline characteristics exhibiting statistical difference were incorporated into LASSO-logistic regression. Six factors were further selected by multivariable logistic regression. The results identified male gender (OR: 2.622; 95% CI: 1.565–4.393, *p* < 0.001), Hb (OR: 0.972; 95% CI: 0.962–0.981, *p* < 0.001), Pro-BNP (OR: 1.000; 95% CI: 1.000–1.000, *p* < 0.001), SIRI (OR: 1.037; 95% CI: 1.026–1.049, *p* < 0.001), and SII (OR: 1.000; 95% CI: 1.000–1.001, *p* = 0.003) as risk factors. These five factors were used to construct a nomogram, which exhibited good accuracy and consistency. **Conclusions:** Male gender, Hb, Pro-BNP, SIRI, and SII were identified as risk factors for CKD risk in HCM patients. A nomogram was developed using these factors, which may facilitate early identification and management of high-risk individuals.

## 1. Introduction

Hypertrophic cardiomyopathy (HCM) is a prevalent genetic heart condition, primarily characterized by abnormal thickening of the left or right ventricle, which leads to alterations in the structure and function of the heart [1,2]. The etiology of HCM is complex and closely related to multiple genetic mutations and environmental factors. Its clinical manifestations are diverse, ranging from asymptomatic cases to heart failure and sudden death, significantly impacting the patient’s quality of life and prognosis. In recent years, deeper investigations into HCM have yielded an increasing body of evidence suggesting that HCM patients bear a high burden from multi-system diseases, particularly exhibiting an increased risk for chronic kidney disease (CKD) [3,4]. Research has indicated that the coexistence of CKD among HCM patients not only elevates the incidence of cardiovascular events but also substantially elevates mortality risk. This comorbid condition presents additional challenges for clinical management of HCM patients, highlighting the critical clinical importance of early prediction and identification of CKD in this population.

It is currently believed that HCM patients exhibit a state of “chronic low-grade” inflammation, characterized by elevated levels of pro-inflammatory cytokines, which may contribute to the progression of HCM and ultimately influence clinical outcomes [5]. Persistent low-grade inflammation is recognized as a critical factor in the development and progression of CKD [6]. The unique physiological function of kidneys render them particularly susceptible to inflammation-induced damage [7]. Complete blood cell count (CBC)-derived inflammatory indicators, such as systemic inflammatory response index (SIRI), can be obtained through routine laboratory examinations and have demonstrated comprehensive efficacy in reflecting chronic systematic inflammation [8]. One study suggested that SIRI serves as a risk factor for all-cause mortality among HCM patients [9]. Another investigation involving 40,937 individuals found that elevated SIRI levels were significantly associated with both the prevalence of CKD and the mortality rate among CKD patients [10]. Collectively, these findings illuminate the promising potential of CBC-derived inflammatory indicators in predicting CKD within HCM patients.

This study aims to explore the role of CBC-derived inflammatory indicators in relation to coexisting CKD among HCM patients, which may assist in identifying those at higher risk for developing CKD. The results may provide valuable insights into the mechanisms linking inflammation with comorbidity such as CKD, thereby improving early detection strategies for CKD in this patients group.

## 2. Materials and Methods

### 2.1. Participants Enrollment

Patients who were first hospitalized at the First Affiliated Hospital of Xi’an Jiaotong University from January 2014 to December 2023 due to HCM were included. Diagnostic criteria for HCM are based on the guideline of the European Society of Cardiology, which mainly include (1) wall thickness ≥ 15 mm in any segments of the left ventricular (LV) myocardium and (2) a condition not solely explained by loading conditions [11]. The exclusion criteria were as follows: (1) missing echocardiography or cardiac magnetic resonance imaging results, which could not confirm the diagnosis; (2) previous medical history of renal disease; (3) missing data which could not be fulfilled by statistical methods.

This study was conducted in accordance with the Declaration of Helsinki and was approved by the Ethics Committee of the First Affiliated Hospital of Xi’an Jiaotong University (XJTU1AF2024LSYY-453; date: 28 November 2024). The Ethics Committee of the First Affiliated Hospital of Xi’an Jiaotong University granted a waiver for informed consent to this study due to its retrospective nature.

### 2.2. Data Collection and Grouping

Basic demographic information, comorbidities, biochemical indicators, and imaging results at admission were extracted from the electronic medical records in The Biobank of the First Affiliated Hospital of Xi’an Jiaotong University. Enrolled patients were separated into the CKD group and non-CKD group according to their discharge diagnoses. The diagnosis of CKD was defined as abnormalities of kidney structure or function persisting for a minimum of 3 months, with implications for health, based on the KDIGO 2024 Clinical Practice Guideline [12].

The CBC-derived inflammatory biomarkers were as follows: SIRI = (neutrophils × monocytes)/lymphocytes; systemic immune inflammation index (SII) = (neutrophils × platelets)/lymphocytes; neutrophil–lymphocyte ratio (NLR) = neutrophils/lymphocytes; and platelet–lymphocyte ratio (PLR) = platelets/lymphocytes.

### 2.3. Statistical Analysis

Variables with less than 20% missing values were managed with multiple interpolation techniques. The continuous variables that followed a normal distribution were presented as mean ± standard deviation and were compared with Student’s *t*-tests. Those not following a normal distribution were represented as median with interquartile range (25th and 75th percentile) and were compared with Mann–Whitney U tests. Categorical variables were reported as absolute counts with relative frequencies. Chi-square tests and Fisher’s exact tests were employed for comparisons. Receiver operating characteristic (ROC) curves with area under curve (AUC) and its 95% confidence interval (CI) were constructed to evaluate the predictive ability of each indicator. The relaxed least absolute shrinkage and selection operator (LASSO) logistic regression model were used to determine the risk factors for CKD, which were subsequently examined by multivariate logistic regression analysis. The independent risk factors identified would be used to develop a nomogram for clinical application. Data analysis was conducted using SPSS software (version 27.0, Chicago, IL, USA) and R software (version 4.1.1, Boston, MA, USA).

## 3. Results

### 3.1. Baseline Characteristics

A total of 1795 HCM patients were included in this study, among which 112 (6.24%) presented with CKD. The baseline characteristics of the two groups were summarized in Table 1. The CKD group exhibited higher proportion of male and patients with coronary artery disease compared to the non-CKD group. Additionally, the CKD group demonstrated elevated levels of white blood cell count, neutrophil count, monocyte count, creatinine, blood urea nitrogen, pro-brain natriuretic peptide (Pro-BNP), creatine kinase isoenzymes MB, and CBC-derived inflammatory indicators compared to the non-CKD group (all *p* < 0.05). Conversely, hemoglobin (Hb), lymphocyte count, alanine aminotransferase, aspartate aminotransferase, albumin, estimated glomerular filtration rate, high-density lipoprotein, and left ventricle ejection fraction were lower in the CKD group in comparison to the non-CKD group (all *p* < 0.05).

### 3.2. Univariate Predictive Effect of CBC-Derived Inflammatory Indicators

**Figure 1:**
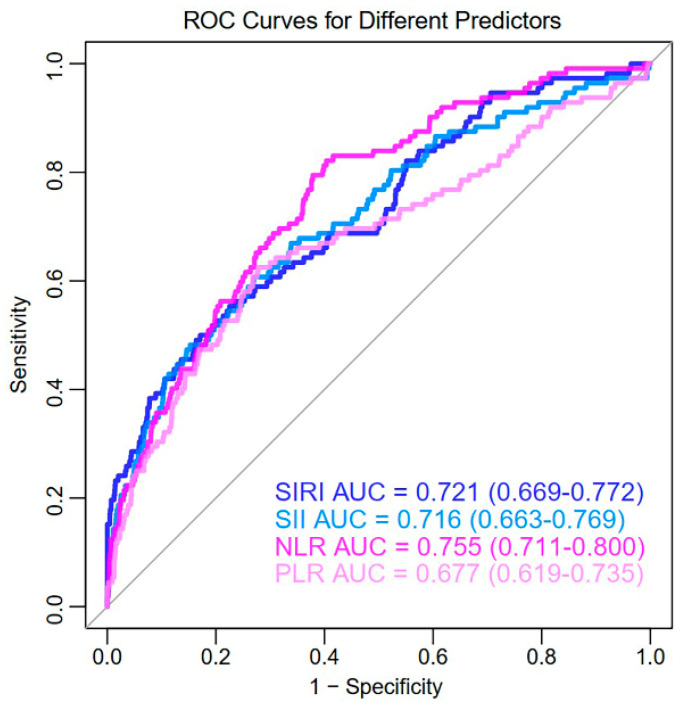
ROC curves of CBC–derived inflammatory indicators in predicting CKD in HCM patients. ROC, receiver operating characteristic curves; SIRI, systemic inflammation response index; SII, systemic immune inflammation index; NLR, neutrophil-to-lymphocyte ratio; PLR, platelet-to-lymphocyte ratio.

The ROC curves of CBC-derived inflammatory indicators are illustrated in Figure 1. NLR (AUC: 0.755; 95%CI: 0.711–0.800) demonstrated superior accuracy in predicting CKD in HCM patients alone, outperforming SIRI (AUC: 0.721; 95%CI: 0.669–0.772), SII (AUC: 0.716; 95%CI: 0.663–0.769), and PLR (AUC: 0.677; 95%CI: 0.619–0.735).

### 3.3. LASSO-Logistic Regression and Multivariate Logistic Regression Analysis

**Figure 2:**
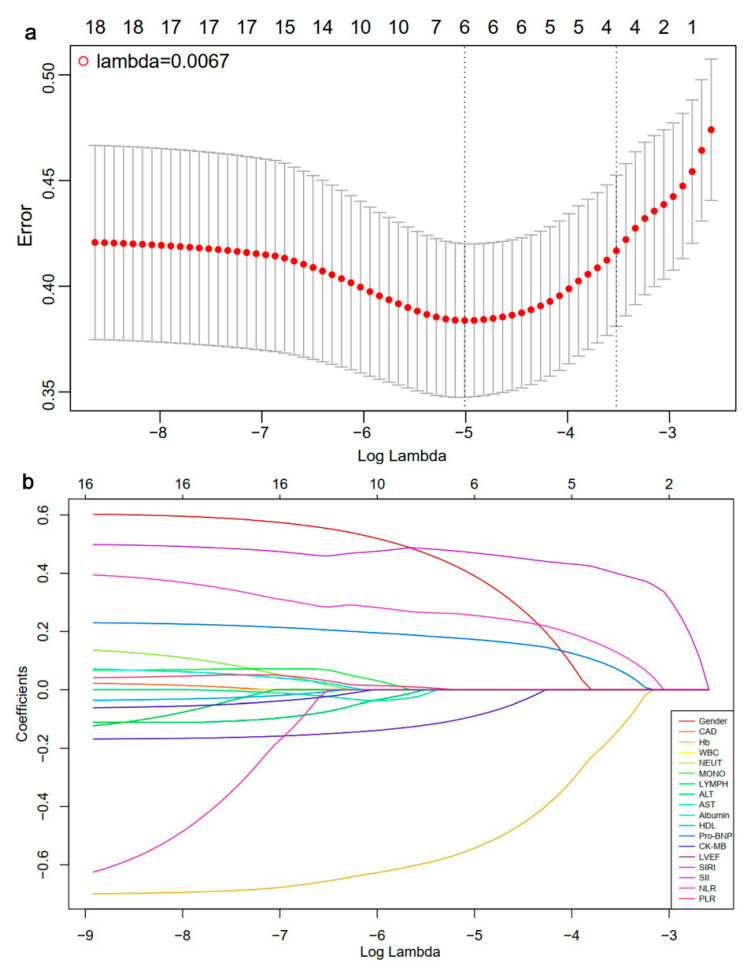
Results of LASSO-Logistic regression: (**a**) the cross-validation plot of LASSO-logistic regression; (**b**) selection process of Lasso regression model by cross-validation method. CAD, coronary artery disease; Hb, hemoglobin; WBC, white blood cell; NEUT, neutrophil count; MONO, monocyte count, LYMPH, lymphocyte count; ALT: alanine aminotransferase; AST, aspartate aminotransferase; HDL, high-density lipoprotein; Pro-BNP, pro-brain natriuretic peptide; CK-MB, creatine kinase isoenzymes MB; LVEF, left ventricle ejection fraction; SIRI, systemic inflammation response index; SII, systemic immune inflammation index; NLR, neutrophil-to-lymphocyte ratio; PLR, platelet-to-lymphocyte ratio.

Given that the estimated glomerular filtration rate, creatinine, and blood urea nitrogen were related to the diagnosis of CKD, they were excluded from further analysis. Other mentioned characteristics with statistical difference at baseline were available for LASSO-logistic regression analysis. The midpoint between the two dotted lines indicates the range for positive and negative standard deviations of log (lambda). When lambda min = 0.0067 and log (lambda min) = −5.0056, six factors were selected for further evaluation (Figure 2, Table 2). After conducting multivariate logistic regression, male gender (OR: 2.622; 95% CI: 1.565–4.393, *p* < 0.001), Hb (OR: 0.972; 95% CI: 0.962–0.981, *p* < 0.001), Pro-BNP (OR: 1.000; 95% CI: 1.000–1.000, *p* < 0.001), SIRI (OR: 1.037; 95% CI: 1.026–1.049, *p* < 0.001), and SII (OR: 1.000; 95% CI: 1.000–1.001, *p* = 0.003) emerged as independent risk factors for CKD in HCM patients (Table 2).

### 3.4. Construction of Nomogram

**Figure 3:**
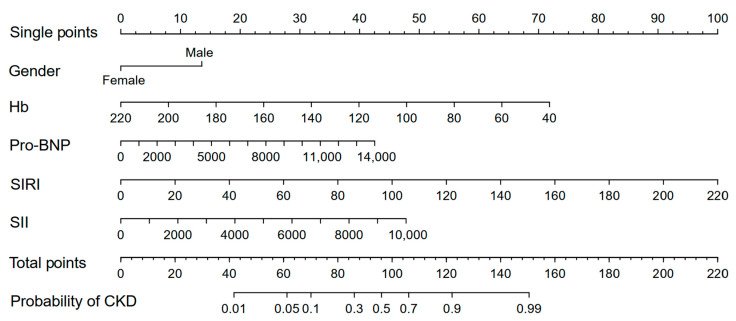
Nomogram to predict the possibility of CKD in HCM patients. HCM, hypertrophic cardiomyopathy; CKD, chronic kidney disease; Hb, hemoglobin; Pro-BNP, pro-brain natriuretic peptide; SIRI, systemic inflammation response index; SII, systemic immune inflammation index.

**Figure 4:**
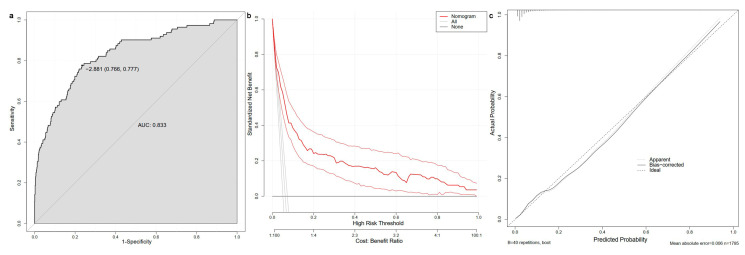
Receiver operating characteristic curve (**a**), decision curve (**b**), and calibration curve (**c**) of the nomogram. AUC, area under curve.

The five risk factors identified through LASSO-logistic and multivariate logistic regression were used to construct a nomogram for predicting the CKD in HCM patients (Figure 3). A single point could be obtained for five factors, depending on biochemical examination and state of patients. The five single points could be added to a total point, which corresponded to the likelihood of CKD. The AUC value of ROC curve was found to be 0.833. Additionally, the decision curve and calibration curve indicated good accuracy and consistency of the nomogram (Figure 4). 

## 4. Discussion

This study enrolled HCM patients and separated them into two groups based on the CKD diagnosis. The findings revealed that NLR is particularly effective in predicting CKD when considered independently. Further LASSO-logistic and multivariate logistic regression defined male gender, Hb, Pro-BNP, SIRI, and SII as independent risk factors for CKD. A nomogram constructed from these five variables exhibited strong accuracy and applicability. This research provides valuable insights into the association between CBC-derived inflammatory indicators and CKD risk among HCM patients, suggesting potential biomarkers for early identification of high-risk individuals and contributing to the understanding of inflammation’s role in disease progression.

HCM is the most common inherited heart disease, characterized by a variety of phenotypes [13]. Previous studies suggest that the divergence of cardiac-specific hypertrophy, inflammation, and fibrosis pathways may be key to understanding the different phenotypes of this condition [14,15]. Johanna Kuusisto et al. found that invasive inflammatory monocytes in tissue samples from patients with HCM undergo cardiac trans endothelial migration, accompanied by an increase in pro-inflammatory cytokine levels, including interleukin-6, and C-reactive protein (CRP) [16]. A study involving 97 cases of HCM found that the levels of NLR were significantly higher in the HCM group compared to the control group [17]. Previous studies have found that elevated hsCRP levels are independently associated with cardiovascular mortality in patients with HCM [18]. CRP may play a pathogenic role in the development of cardiac hypertrophy and fibrosis, possibly through the activation of the nuclear factor kappa B (NF-κB) signaling pathway [19]. Rongxin Zhang et al. also found that the activation of the NF-κB pathway could mediate the pro-fibrotic effects of CRP, potentially contributing to the progression of cardiac fibrosis [20]. NF-κB, a key regulator of inflammation, is known to induce the expression of various pro-inflammatory cytokines and extracellular matrix components, which are critical in the development of fibrosis. In the context of cardiac hypertrophy, NF-κB activation can promote the transformation of cardiac fibroblasts into myofibroblasts, leading to excessive deposition of collagen and other extracellular matrix proteins, ultimately resulting in myocardial fibrosis. This process is considered a precursor to hypertrophic remodeling in conditions such as HCM. Myocardial fibrosis in HCM is not only a hallmark of the disease but also represents an early stage of pathological remodeling before overt hypertrophy becomes apparent. Myocardial fibrosis is a key determinant of adverse cardiovascular events such as sudden cardiac death (SCD), ventricular arrhythmias, LV dysfunction, and heart failure [21]. In HCM patients, inflammation is closely associated with poor prognosis. The chronic inflammatory response in these patients contributes to the progression of myocardial fibrosis, which disrupts normal heart structure and function. Inflammation accelerates pathological remodeling, increases the risk of arrhythmias, and worsens heart failure, thereby leading to worse clinical outcomes in HCM patients [22,23]. The persistent inflammatory state in HCM may thus serve as a critical factor in the development of adverse events.

Kidneys are a key organ that interacts with the heart in systemic physiology. Previous studies have found that CKD is a significant risk factor for increased cardiovascular morbidity and mortality, with outcomes worsening in proportion to the degree of renal dysfunction [24,25]. Among patients with end-stage renal diseases, approximately 50% of deaths are attributed to cardiovascular disease. Pressure overload related to cardiac hypertrophy can lead to a serious of pathophysiological changes, including mitochondrial dysfunction, dysregulation of Ca^2+^-handling proteins and metabolic changes, which may directly or indirectly affect kidney function [26]. Meanwhile, various cardiac conditions, including HF, can exacerbate renal function decline. Due to the progressive clinical or subclinical heart failure, primarily attributed to LV diastolic dysfunction and systolic dysfunction, heart failure represents one of the most important complications of HCM, leading to long-term adverse effects on kidney function [27,28,29,30]. A study involving 10,300 patients with HCM found that compared to the control group, the incidence of end-stage kidney disease (ESKD) during follow-up was significantly higher in HCM patients (4.14/1000 vs. 0.60/1000) [31]. The pathogenesis of CKD in HCM is likely multifactorial. One potential mechanism involves diastolic dysfunction in HCM, which leads to a stiff LV. This stiffness may trigger activation of the renin–angiotensin system, resulting in elevated LV filling pressures and renal venous pressure. These changes can promote unfavorable fluid redistribution, ultimately impairing renal function [32].

The role of inflammation in the initiation and progression of various cardiovascular diseases has been extensively investigated. It is also considered a significant factor contributing to poor prognostic outcomes and complications of cardiovascular diseases [33]. CBC-derived inflammation biomarkers are a serious of indicators that take monocytes, neutrophils, lymphocytes, and platelets into account. Although NLR indicated superior accuracy in predicting CKD alone, previous studies have shown that SIRI is more reliable and representative of inflammation than traditional markers such as PLR and NLR. SIRI may serve as a comprehensive indicator for predicting coagulation and inflammation risks in cardiovascular disease (CVD) events [34]. The values of SIRI were influenced by monocytes, neutrophils, and lymphocytes. Among them, monocytes can infiltrate tissues and differentiate into macrophages, playing a key role in immune defense and tissue repair [35]. Monocytes also play a key role in CKD progression, with the NLRP3 inflammasome activated by stimuli like reactive oxygen species (ROS), leading to IL-1 release and the activation of pro-inflammatory pathways such as NF-kB and AP-1. This disrupts renal units and causes microvascular inflammation. Chronic myocardial stress and inflammation can also exacerbate systemic inflammation in HCM patients. Elevated SIRI levels in HCM patients may amplify this inflammatory response, increasing the risk of CKD through a shared inflammatory mechanism, linking cardiac and renal dysfunction [36,37]. Moreover, neutrophils can contribute to CKD progression through mechanisms such as the secretion of neutrophil serine proteases and the release of neutrophil extracellular traps. These inflammatory processes may also contribute to cardiac and renal dysfunction through the heart–kidney axis [10]. In addition, this study found that Hb has clinical value in differentiating HCM patients with CKD. One study revealed that Hb levels are inversely proportional to LV outflow tract pressure, which is significant for predicting outflow tract obstruction in HCM patients [38]. It is speculated that individuals with concurrent outflow obstruction may be more susceptible to complications such as CKD. Generally, as CBC-derived inflammation indicators were convenient to obtain, they have great potential in reflecting the inflammation levels, building a link between diseases.

However, this study has several limitations. First, its retrospective design may introduce biases, limiting causal inference capabilities. Second, being conducted at a single center restricts the generalizability of our findings to broader populations. Furthermore, the absence of longitudinal follow-up precludes an evaluation of the long-term effects associated with elevated SIRI on CKD progression.

## 5. Conclusions

In conclusion, this study demonstrates that NLR was associated with an increased risk of CKD in HCM patients when assessed independently. Following LASSO-logistic and multivariate logistic regression, male gender, Hb, Pro-BNP, SIRI, and SII collectively present potential clinical implications in the assessment CKD risk within the HCM population. This finding highlights the role of CBC-derived inflammation biomarkers as predicters for CKD risk in HCM patients. Additionally, the interplay between the heart and kidneys in HCM may create a “vicious cycle” that accelerates CKD progression, which deserves more clinical attention.

## Figures and Tables

**Table 1:** Baseline characteristics between the two groups.

Items	Total (*n* = 1795)	CKD Group (*n* = 112)	Non-CKD Group (*n* = 1683)	*p*-Value
Age (years)	61 (51, 69)	61 (49, 70)	61 (51, 68)	0.901
Gender (male, %)	1184 (66.0)	85 (75.9)	1099 (65.3)	0.022
BMI (kg/m^2^)	24.95 ± 3.51	25.09 ± 4.05	24.94 ± 3.47	0.723
Smoking (%)	723 (40.3)	51 (45.5)	672 (39.9)	0.241
Drinking (%)	217 (12.1)	15 (13.4)	202 (12.0)	0.662
Comorbidities				
Hypertension (%)	835 (46.5)	58 (51.8)	777 (46.2)	0.248
Diabetes (%)	250 (13.9)	22 (19.6)	228 (13.5)	0.071
CAD (%)	92 (5.1)	12 (10.7)	80 (4.8)	0.006
Biochemical results				
Hb (g/L)	140 (127, 152)	129 (108, 145)	141 (128, 153)	<0.001
WBC (10^9^/L)	6.53 (5.28, 8.42)	7.42 (5.67, 10.88)	6.46 (5.27, 8.31)	<0.001
NEUT (10^9^/L)	4.07 (3.23, 5.38)	5.41 (3.73, 7.41)	4.04 (3.21, 5.29)	<0.001
MONO (10^9^/L)	2.90 (0.37, 6.00)	5.10 (0.69, 6.88)	1.07 (0.37, 5.90)	<0.001
LYMPH (10^9^/L)	1.50 (1.13, 1.91)	1.14 (0.77, 1.53)	1.52 (1.16, 1.93)	<0.001
PLT (10^9^/L)	188 (152, 230)	182 (147, 245)	188 (153, 229)	0.815
ALT (U/L)	22.00 (15.50, 34.00)	20.00 (14.00, 30.75)	22.40 (16.00, 34.00)	0.030
AST (U/L)	24.00 (19.70, 31.00)	22.00 (17.00, 28.75)	24.00 (20.00, 31.00)	0.003
Albumin (g/L)	39.70 (36.70, 42.70)	36.8 (32.65, 41.93)	39.90 (37.00, 42.70)	<0.001
eGFR (mL/min/1.73 m^2^)	96.13 (82.56, 106.71)	43.48 (22.37, 68.36)	97.11 (85.89, 107.57)	<0.001
Cr (umol/L)	68 (55, 80)	127 (96, 227)	67 (55, 78)	<0.001
BUN (mmol/L)	5.96 (4.94, 7.31)	9.53 (6.88, 15.08)	5.89 (4.88, 7.15)	<0.001
HDL (mmol/L)	0.97 (0.81, 1.14)	0.90 (0.74, 1.11)	0.97 (0.82, 1.14)	0.025
LDL (mmol/L)	2.17 (1.66, 2.75)	1.99 (1.50, 2.65)	2.18 (1.67, 2.76)	0.104
TG (mmol/L)	1.29 (0.93, 1.81)	1.37 (0.87, 1.81)	1.29 (0.93, 1.81)	0.775
TC (mmol/L)	3.83 (3.22, 4.49)	3.76 (3.19, 4.36)	3.84 (3.24, 4.52)	0.467
Pro-BNP (pg/mL)	1141 (362, 2887)	2931 (999, 7135)	1069 (342, 2710)	<0.001
LDH (U/L)	225 (198, 263)	243 (198, 287)	225 (197, 261)	0.069
CK-MB (U/L)	13.69 (10.00, 18.09)	14.85 (10.85, 20.90)	13.50 (10.00, 18.00)	0.049
CK (U/L)	27.80 (12.34, 79.00)	33.00 (13.78, 65.00)	27.10 (12.21, 79.00)	0.873
Echocardiology				
LVEDD (mm)	48 (45, 52)	48 (44, 52)	48 (45, 52)	0.770
LVESD (mm)	29 (26, 32)	30 (26, 32)	29 (26, 32)	0.079
IVST (mm)	14 (11, 18)	15 (11, 18)	14 (11, 18)	0.139
LVEF (%)	68 (63, 73)	66 (58, 71)	68 (63, 73)	0.001
CBC-Derived inflammatory indicators				
SIRI	6.68 (0.99, 16.66)	19.27 (3.25, 38.11)	5.96 (0.96, 15.86)	<0.001
SII	501.15 (345.31, 766.65)	855.74 (497.43, 1609.18)	488.21 (340.61, 736.79)	<0.001
NLR	2.68 (1.96, 3.83)	4.26 (3.14, 7.48)	2.61 (1.93, 3.70)	<0.001
PLR	125.00 (94.25, 166.67)	174.37 (112.68, 259.18)	123.33 (93.55, 163.00)	<0.001

CKD, chronic kidney disease; BMI, body mass index; CAD, coronary artery disease; Hb, hemoglobin; WBC, white blood cell; NEUT, neutrophil count; MONO, monocyte count, LYMPH, lymphocyte count; PLT, platelet; ALT: alanine aminotransferase; AST, aspartate aminotransferase; eGFR, estimated glomerular filtration rate, Cr, creatinine; BUN, blood urea nitrogen; HDL, high-density lipoprotein; LDL, low-density lipoprotein; TG, triglycerides; TC, total cholesterol; Pro-BNP, pro-brain natriuretic peptide; LDH, lactate dehydrogenase; CK-MB, creatine kinase isoenzymes MB; CK, creatine kinase; LVEDD, left ventricular end-diastolic diameter; LVESD, left ventricular end-systolic diameter; IVST, interventricular septal thickness; LVEF, left ventricle ejection fraction; CBC, complete blood cell count; SIRI, systemic inflammation response index; SII, systemic immune inflammation index; NLR, neutrophil-to-lymphocyte ratio; PLR, platelet-to-lymphocyte ratio.

**Table 2:** Results pf LASSO-logistic regression and multivariate regression model.

Items	LASSO-Logistic Regression		Multivariate Logistic Regression	
	Assignment	Coefficient	OR (95% CI)	*p*-Value
Gender (Male, %)	Male = 1; Female = 0	0.5988	2.622 (1.565–4.393)	<0.001
CAD (%)	Yes = 1; No = 0			
Hb (g/L)	Continuous variable (reference range: 115–150)	−0.6793	0.972 (0.962–0.981)	<0.001
WBC (10^9^/L)	Continuous variable (reference range: 3.5–5.5)			
NEUT (10^9^/L)	Continuous variable (reference range: 40–75)			
MONO (10^9^/L)	Continuous variable (reference range: 0.1–0.6)			
LYMPH (10^9^/L)	Continuous variable (reference range: 1.1–3.2)			
ALT (U/L)	Continuous variable (reference range: 7–40)			
AST (U/L)	Continuous variable (reference range: 13–45)			
Albumin (g/L)	Continuous variable (reference range: 40–55)			
HDL (mmol/L)	Continuous variable (reference range: 1.16–1.42)			
Pro-BNP (pg/mL)	Continuous variable (reference range for healthy populations: 0–125)	0.2029	1.000 (1.000–1.000)	<0.001
CK-MB (U/L)	Continuous variable (reference range: 0–24)			
LVEF (%)	Continuous variable	−0.1696	0.983 (0.964–1.003)	0.095
SIRI	Continuous variable	0.5143	1.037 (1.026–1.049)	<0.001
SII	Continuous variable	0.2917	1.000 (1.000–1.001)	0.003
NLR	Continuous variable			
PLR	Continuous variable			

LASSO, least absolute shrinkage and selection operator; OR, odds ratio; CI, confidence interval. Other abbreviations as in Table 1.

## Data Availability

The data underlying this article cannot be shared publicly due to the privacy of individuals that participated in the study. The data will be shared on reasonable request to the corresponding author.

## Abbreviations

HCM	Hypertrophic cardiomyopathy
CKD	Chronic kidney disease
LV	Left ventricular/ventricle
CBC	Complete blood cell counts
SIRI	Systemic inflammatory response index
SII	Systemic immune inflammation index
NLR	Neutrophil–lymphocyte ratio
PLR	Platelet–lymphocyte ratio
ROC	Receiver operating characteristic
AUC	Area under curve
CI	Confidence interval
LASSO	Least absolute shrinkage and selection operator
Pro-BNP	Pro-brain natriuretic peptide
Hb	Hemoglobin
